# Knowledge and attitude to HIV pre exposure prophylaxis among women in five sub-Saharan African countries: a multilevel model analysis of population-based survey 2021–2022

**DOI:** 10.1186/s12889-024-18717-1

**Published:** 2024-05-07

**Authors:** Bewuketu Terefe, Mahlet Moges Jembere, Dejen Kahsay Asgedom, Ayenew Molla Lakew

**Affiliations:** 1 Department of Community Health Nursing, School of Nursing, College of Medicine and Health Sciences, University of Gondar, Gondar, Ethiopia; 2 Department of Emergency, and Critical Care Nursing, School of Nursing, College of Medicine and Health Sciences, University of Gondar, Gondar, Ethiopia; 3 Department of Public Health, College of Medicine and Health Sciences, Samara University, Afar, Ethiopia; 4 Department of Epidemiology, and Biostatistics, Institute of Public Health, College of Medicine and Health Sciences, University of Gondar, Gondar, Ethiopia

**Keywords:** Knowledge, Attitude, HIV pre exposure prophylaxis, Women, Sub-Saharan africa

## Abstract

**Background:**

HIV Pre-Exposure Prophylaxis (HIV PrEP) may help reduce the rate of HIV infection among women in sub-Saharan Africa (SSA). This study aimed to assess women’s knowledge and attitudes toward PrEP, a crucial component of HIV prevention, using nationwide data. It is the first study of its kind conducted in five SSA countries: Burkina Faso, Ghana, Côte d’Ivoire, Kenya, and Tanzania. The primary objective was to examine women’s knowledge and attitudes toward PrEP for the prevention of HIV infection, as well as to explore individual- and community-level factors associated with it.

**Methods:**

The current study utilized the 2021/22 demographic and health survey datasets from five African nations, namely Burkina Faso, Côte d’Ivoire, Ghana, Kenya, and Tanzania. The analysis was performed using Stata 17. A weighted sample of 77,052 women of reproductive age participated in the survey. Univariate and multivariable multilevel logistic regressions were conducted to assess parameters related to knowledge and attitudes toward PrEP in these countries. In both the univariate regression and the final model, the significance of variables was determined using P values of ≤ 0.2 and < 0.05.

**Results:**

Overall, only about 13.88 (95% CI: 13.64,14.12) of women had knowledge and attitudes toward HIV PrEP. The highest (34.29%) and lowest (5.61%) values were observed for Kenya and Tanzania respectively. Higher rates of knowledge, and attitude toward HIV PrEP among women were independently associated with age 25–34 years old (AOR = 1.52, 95% CI:1.41,1.64), and 35–49 years old (AOR = 1.56, 95% CI:1.43,1.69), primary education level (AOR = 1.79,95% CI:1.65,1.95), and secondary/higher education level (AOR = 2.92, 95% CI: 2.67,3.20), richer (AOR = 1.14, 95% CI:1.02,1.27), and richest (AOR = 1.21, 95% CI:1.06,1.37), employed women (AOR = 1.82, 95% CI:1.65,1.99), had media exposure (AOR = 1.49,95% CI:1.40,1.59),knowledge of modern contraception (AOR = 2.62, 95% CI: 1.94,3.43), had at least one ANC visit (AOR = 1.99, 95% CI:1.47,2.69), gave birth at health institutions (AOR = 1.17, 95% CI: 1.02,1.37), ever had given birth (AOR = 1.53, 95% CI: 1.41,1.66), female household heads (AOR = 1.24, 95% CI:1.17,1.31), rural women (AOR = 0.83, 95% CI: 0.76,0.89). Similarly, women from communities with high ANC coverage (AOR = 1.84, 95% CI: 1.61,2.11), high community mass media exposure (AOR = 1.62, 95% CI: 1.39,1.88), and high community wealth level (AOR = 1.48, 95% CI: 1.30,1.68), and women from the high illiteracy rate community (AOR = 0.71, 95% CI: 0.61,0.82) showed statistically significant associations with the outcome variable in the final model.

**Conclusions:**

Less than one-seventh of women exhibited knowledge of and positive attitudes toward HIV PrEP. All stakeholders involved in HIV/AIDS prevention and control have recognized the significance of the factors mentioned above. Enhancing maternal health services, such as promoting institutional delivery, contraception, antenatal care (ANC), and women’s empowerment, alongside harnessing the power of media and embracing these transformative changes, will contribute to a greater understanding of and more favorable attitudes toward HIV PrEP within the population.

## Introduction

Globally, HIV/AIDS is a significant public health concern affecting communities. With an estimated 38.4 million (33.9–43.8 million) people living with HIV in 2022, 53% of them being women and girls, 1.5 million children, 1.5 million new infections (ranging from 1.1 to 2.0 million) that year, and 650,000 AIDS-related deaths (ranging from 510,000 to 860,000) in 2021, the global HIV numbers are alarming. Furthermore, Sub-Saharan Africa (SSA) bears the highest burden of the HIV epidemic, accounting for more than two-thirds of the cases [1–4]. In SSA, women experience a disproportionately high prevalence of HIV, despite the already elevated overall risk of HIV infection in the population. This means that women in SSA face a greater burden of HIV compared to other demographic groups, highlighting the urgent need for targeted interventions and strategies to address this disparity [5, 6]. Although they constitute a smaller proportion of the overall population, adolescent girls and young women account for approximately one in three new infections [6, 7]. Compared to their male counterparts, women in SSA are five times more likely to be living with HIV and have contracted the virus five to seven years earlier [6–8]. Various biological, social, cultural, behavioral, economic, and structural variables contribute to this heavy burden [9, 10]. In terms of social factors, the deterioration or inequality in education, poverty, unemployment, and access to other social services can lead to a loss of opportunities for HIV prevention, particularly among women. Behavioral factors, including abstinence or delaying sexual debut, improper condom use, engaging in unsafe or forced sex, practicing polygamy, increasing the number of sexual partners, and not participating in voluntary counseling and testing, also play a significant role [10–12]. Additionally, being asymptomatic for sexually transmitted infections (STIs) poses a challenge for HIV infection prevention among women in SSA. Biological factors like this contribute to the complexity of addressing HIV transmission in the region [10, 13]. Furthermore, according to the UNAIDS analysis of HIV prevalence in Kenya (2018), women account for 65% of the HIV-positive population, and the incidence among young women (11,000) is more than twice that among young men (5000) [14].

Regarding the incidence of HIV infection Pre-exposure prophylaxis (PrEP) has been shown to have significant promise for significantly reducing HIV transmission among important populations worldwide [15]. Hence, the World Health Organization (WHO) advises including PrEP in HIV prevention packages for women living in high-burden environments [16]. The uptake of PrEP among women in SSA remains low, despite a significant percentage of women being vulnerable. However, there is a lack of evidence to support further investigation in this area. Additionally, a WHO report indicates that while there is increasing recognition of the potential of PrEP as an additional HIV prevention strategy, many countries are still in the early stages of exploring effective implementation methods. Moreover, the availability of PrEP outside of research and demonstration projects in lowand middle-income countries has been limited, resulting in a lack of practical experience in its provision [17].

When PrEP is used as directed, it significantly reduces the risk of contracting HIV through sexual activity by 99% and through injectable drug use by 74%) [18, 19]. Other studies have shown that when taken daily, PrEP prevents HIV acquisition in women by > 90% [20, 21]. However, in SSA, the implementation of PrEP involves several multidimensional challenges. These obstacles can be divided into three categories: patient-related, clinical, and community. Patient-level obstacles include misperceptions of risk, concerns about side effects, and lack of knowledge regarding PrEP [22, 23]. Clinic-level obstacles include inadequate patient communication, overworked personnel, excessive caseloads, and limited clinician competence [23–25]. Stigma around premarital sex; stigma associated with HIV; restricted access to PrEP outside medical institutions; and a lack of support from partners, parents, and community members who could have an impact on a patient’s decision-making are examples of community-level hurdles [23, 26, 27]. Challenges are continuing in developing countries, particularly in SSA.

There has been modest progress in overcoming obstacles to PrEP implementation, such as start and adherence, in countries where the PrEP initiative has been implemented [28, 29]. These successes are attributed to recognizing the needs of young women and girls within the context of their local communities, emphasizing their empowerment, and addressing institutional obstacles. However, there is a lack of nationally representative data to examine women’s attitudes and knowledge levels in these nations, and this study represents the first of its kind in SSA. One of the significant objectives of this study is to bridge the knowledge gap by exploring factors, empirical evidence, populations, and up-to-date information for relevant stakeholders. Monitoring the uptake, adherence, and retention of PrEP programs is crucial for scaling up treatment in SSA countries through national health systems. Specifically, our focus was on the five countries included in the Demographic and Health Survey (DHS) report from 2021 to 2022, as they were the only SSA countries with available data on HIV PrEP knowledge and attitudes among women. Furthermore, gaining insights into the factors that influence patients’ decisions to initiate PrEP will contribute to the development of more affordable PrEP therapies and enhance HIV prevention efforts in the region. Therefore, using data from the most recent nationwide health survey, this study aimed to investigate women’s knowledge and attitudes, considering both individual- and community-level characteristics, in five African countries.

## Methods

### Study setting and period

Data were collected between 2021 and 2022 for a nationwide cross-sectional study conducted in communities across five SSA countries: Burkina Faso, Côte d’Ivoire, Ghana, Kenya, and Tanzania. The study utilized the DHS program, which is funded by the United States Agency for International Development (USAID) and provides financial and technical assistance for demographic and health surveys in countries worldwide. The data used in this study were obtained from the most recent DHS dataset for SSA countries in the past couple of years (2021–2022). The DHS program ensures the use of standardized data collection methods across all countries [30]. In order to ensure a sizable and representative sample size that accounts for various factors, the DHS conducts surveys on a global scale. These surveys are designed to gather comparable data. They employ large sample sizes and are population-based, aiming to provide nationally representative information for each country [30].

### Data source and study population

The DHS, conducted between 2021 and 2022, served as the data source for this study. Although more than 35 SSA nations conducted the DHS, only survey data from approximately five countries were utilized in this analysis. The selection was made based on the availability of data on the outcome variable, specifically HIV PrEP. Therefore, the data on HIV PrEP were included only from Burkina Faso, Ghana, Côte d’Ivoire, Kenya, and Tanzania in the survey. These were the only five countries that reported information on PrEP regarding HIV in their DHS reports at the time of the study. After incorporating the data from each nation, the final analysis was conducted using a weighted sample of 77,052 women of reproductive age and an unweighted sample of 76,608 women who were asked about their knowledge and attitude toward HIV PrEP for preventing HIV infection. Proportional representation of women was ensured across each country, as shown in Table 1. The individual records (IR) from the DHS databases were utilized for this study. Prior to using the DHS dataset, weighted values were applied to restore the representativeness of the sample data. This is necessary because the probability of selecting a household is not constant throughout the sampling process. The DHS employs five sampling weighting methods, one of which is used for women (v005). Individual sample weights were calculated by dividing the value v005 by 1,000,000. These weights were then utilized to estimate the number of cases in the analysis [31].

**Table 1:** Countries, survey years, and sample size of demographic and health surveys included in the analysis for five Sub Saharan African countries, 2021–2022

Country	Survey year	Sample size (weighted)	Frequency (weighted)
Burkina Faso	2021	17,659	22.92
Côte d’Ivoire	2021	13,221	17.16
Ghana	2022	14,280	18.53
Kenya	2022	16,638	21.59
Tanzania	2022	15,254	19.80
Total		77,052	100

### Sample size determination and sampling procedures

On average, every five years, a systematic compilation of DHS surveys is conducted in low- and middle-income countries. These surveys utilize pretested, validated, and structured questionnaires. The DHS surveys follow a uniform methodology for sampling, questionnaires, data collection, and coding, making it feasible to conduct multi-country analyses. In all the listed countries, the surveys used the most recent conventional census frame as a reference. The DHS samples are typically divided between urban and rural areas within each administrative geographic region.

The DHS employs a stratified two-stage cluster sampling technique. In the first stage, enumeration areas (EAs) and clusters are randomly selected from the sample frame. EAs and clusters are usually created based on the most recent national census. During the initial sample cycle, EAs were chosen with a probability proportional to the size of each stratum. In the second stage, a systematic sampling approach is used to select a predefined number of households within the identified EAs. Equal probability systematic sampling is employed to choose a specific number of households within the designated cluster after the households have been listed [30].

### Data quality control

Before any data were collected, a pre-test was carried out in the DHS. Field workers who had participated in the pre-test were debriefed, and any necessary modifications to the questionnaires were made. Further information on the data-gathering procedure can be found in the DHS guidelines. These data are available in the DHS Statistics Guide [30].

### Data management processes, and analysis

STATA 17 and Microsoft Excel 2019 were used to merge data from the five SSA countries. The sample was then recoded and analyzed, with weighting used to ensure that the overall sample resembled the nation’s actual population and to obtain a trustworthy standard error or statistical estimate. Cross-tabulations and calculations of frequencies and percentages were used in the descriptive analysis. Women belonging to the same cluster resemble one another more than women belonging to separate groups [32]. This results in falsely “significant” results due to overly modest estimated standard errors. A multiple multilevel logistic regression model can account for the lack of independence across nested data layers [33, 34].

Two-stage multivariable multilevel logistic regression models were employed to determine the individual- and community-level factors for women’s HIV PrEP knowledge and attitudes toward preventing HIV among women. Five models were used in this multilevel study. In the first or null model, there was an outcome variable without any independent variable; in the second, there were only individual-level variables; in the third, there were only community-level variables; and in the fourth, there were both individual- and community-level variables. Adjusted odds ratios (AORs) with 95% confidence intervals (CIs) were used to present the modified effects results. Statistical significance was defined as a P value of less than 0.05. The median odds ratio (MOR), proportionate change in variance (PCV), intra-cluster correlation (ICC), log-likelihood ratio, and deviance are five ways in which random effects evaluate variation in women’s HIV PrEP knowledge and attitude to prevent HIV infection across clusters. Deviance information criteria (DIC) and Akaike’s information criterion (AIC) were used to compare models. A model with lower DIC and AIC values was deemed more accurate [35]. Before proceeding to the analysis, each dependent variable was assessed for variance, inflation factor, and tolerance.

### Variables of the study

#### Outcome variable

The dependent variable in this study was women’s knowledge and attitudes regarding HIV PrEP, which is a preventive measure against HIV infection. The study assessed participants’ knowledge and attitudes towards HIV PrEP as the outcome of interest. Participants were asked about their attitudes and the extent of their knowledge regarding the outcome variable. Specifically, they were queried about whether they had never heard of it, heard about it, heard and approved of taking it daily, heard but did not approve of taking it the day before, or heard but were unsure about approving it. For the purpose of this study, women who demonstrated familiarity with HIV PrEP and expressed a positive attitude towards it were considered knowledgeable and had favorable attitudes regarding HIV PrEP. These participants were recoded as “Yes = 1”, while those who did not meet these criteria were recoded as “No = 0”. It is important to note that there were no missing values or unknown variables in this study.

#### Independent variables

Similarly, regarding the explanatory variables like maternal age, marital status, number of under-five, number of sex partner, number of health visits in the past 12 months, current breastfeeding status, maternal education, wealth index, heard about STIs, employment status, mass media exposure, ANC visit, place of delivery, gave birth, sex of the household, residence, community level ANC coverage, community illiteracy, community wealth, community institution delivery coverage, and community mass media exposure were included as an independent factors for knowledge, and attitude to HIV PrEP (Table 2).

**Table 2:** Shows the lists and classifications of independent variables among women in five sub–Saharan African countries

Variables	Categorization
Maternal age	Maternal age was classified as 15–24, 25–34 and 35–49 years old
Marital status	Marital status was categorized as never married, married, and divorced/widowed
Maternal education	Maternal education was classified as no formal education, primary education, and secondary/ high educational level
Wealth	Household wealth index was classified as poorest, poorer, middle, richer and richest respectively
Heard about STIs	Women were asked if they had received information about sexually transmitted infections, with ‘yes’ and ‘no’ options.
Maternal employment	Maternal employment status is classified as ‘yes’ if the woman is involved in any work activity, and ‘no’ if she is not
Mass media exposure	If a woman is exposed to at least one of the three activities, namely watching television, listening to the radio, or reading magazines/books, it is coded as ‘yes’; otherwise, it is coded as ‘no’
Knowledge modern contraceptive method	Knowledge about modern contraceptive methods was categorized as ‘yes’ if the woman knows about them, and as ‘no’ if she does not have knowledge about them
Number of health facility visit in 12 months	The number of health facility visits in the past 12 months was classified as ‘yes’ if the woman visited a health facility at least once, and as ‘no’ if she did not visit any health facilities
At least one ANC visit	The number of antenatal care visits was classified as ‘yes’ if the woman had at least one antenatal care visit during her pregnancy, and as ‘no’ if she did not have any antenatal care visits
Place of delivery	The place of delivery for the recent child was classified as ‘home’ if the woman delivered in her own home or in a relative’s home, and as ‘health facility’ if she gave birth in any health facility, whether it be a public or private institution
Gave birth	This variable was coded as ‘yes’ if the woman has given birth to at least one child in her life, and as ‘no’ if she has not given birth to any children
Number of under five children	The number of under five children in a household was estimated as ‘yes’ if there is at least one child under the age of five in the household, and as ‘no’ if there are no children under the age of five
Current breastfeeding status	If the woman is currently breastfeeding, it is coded as ‘yes’; otherwise, it is coded as ‘no’
Number of sex partner	The number of a woman’s sexual partners was classified as ‘one’ if she had only one partner, and as ‘more than one’ if she had multiple partners
Household head	The household head was classified as ‘male’ if the household is led by a male head, and as ‘female’ if it is led by a female head
Residence	The types of residence were classified as ‘urban’ and ‘rural’
Community ANC	The community-level antenatal care coverage for women was classified as ‘low’ if the coverage was below the median, and ‘high’ if the coverage was equal to or higher than the median in the given community
Community institutional delivery coverage	The community-level institutional delivery coverage for women was classified as ‘low’ if the institutional delivery coverage was below the median, and ‘high’ if the coverage was equal to or higher than the median in the given community
Community mass media	The community-level mass media exposure for women was classified as ‘low’ if the coverage was below the median, and ‘high’ if the coverage was equal to or higher than the median in the given community.
Community wealth	The community-level women wealth index for women was classified as ‘low’ if the coverage was below the median, and ‘high’ if the coverage was equal to or higher than the median in the given community
Community illiteracy	The community-level illiteracy rate for women was classified as ‘high’ if the education rate was below the median, and ‘low’ if the rate was equal to or higher than the median in the given community

### Operational definitions for community level variables

Since the community-level components could not be directly observed or recorded as aggregated data during the survey, all components were estimated using aggregate values derived from the individual records. Each component was estimated by considering the values of distinct variables, following a methodology similar to other studies. In this study, a cluster or primary sample unit in the dataset, shared by a group of families, was considered as a community-level factor. To create variables at the individual, group, and community levels, various components were combined. Community-level variables included community women’s education, media exposure, poverty level, ANC utilization, and place of delivery. Continuous community-level variables were further categorized into low and high groups using the mean or median values based on their distribution. This categorization was done to facilitate a better understanding of the results [36–38].

#### Community’ women’s education

The significance of community women’s educational achievement as a whole is demonstrated by the community’s median distribution of educational attainment. It was considered low if the proportion of women in the community with at least a secondary education fell below the median (50%); it was considered high if it rose beyond the median (51-100%).

#### Community ANC coverage

This variable is derived from individual ANC usage levels. It was classified as high if the percentage of women in the community who attended at least one ANC visit was between 50.3 and 100% and low if the percentage was between 0 and 50.2%.

#### Community health institutional delivery coverage

This variable is derived from each woman’s institutional delivery usage level. It was classified as high if the percentage of women in the community who delivered their last birth in health facilities was between 74.5 and 100% and low if the percentage was between 0 and 74.6%.

#### Community women’s media exposure

Individual responses to media exposure via radio, books, or television served as the basis for this media exposure variable. If the proportion of women in the community who were exposed to media was between 0 and 49%, it was considered low; if it was between 50% and 100%, it was considered high.

#### Community women’s wealth

The same process was used to derive this variable from each household’s wealth index. In a community’s two lowest quintiles of wealth, it was deemed high if there were between 49% and 100% women and low if there were between 0% and 48% of the given household.

## Results

### Sociodemographic characteristics of the study participants

This study involved 77,052 weighted women of reproductive age from five countries. Among the study participants, approximately 29,287 (38.01%) were between 15 and 24 years old. The majority of women, specifically 33,941 (44.05%), were married. In terms of place of residence, 42,298 (54.90%) lived in rural areas, while educational status revealed that 34,198 (44.38%) had attained secondary or higher education. Furthermore, 19,421 (25.20%) belonged to the richest households based on the wealth index, and 60,895 (79.03%) were currently breastfeeding their children. Approximately 46,165 (59.91%) women were employed at the time of the study. Regarding maternal health services, 68,881 (98.36%) had attended at least one antenatal care (ANC) visit, 73,809 (95.79%) had given birth at health institutions, 74,723 (96.98%) were knowledgeable about modern contraceptive methods, and 51,002 (66.19%) had heard about STIs through various channels. Furthermore, 47,974 (62.26%) women had mass media exposure, which involved listening to the radio, watching television, or reading magazines or newspapers. In contrast, almost half of the women, 49,203 (63.86%), reported having more than one sex partner (Table 3).

**Table 3:** Sociodemographic, and maternal related factors on knowledge and attitude of HIV PrEP among women in five Sub Saharan African countries 2021–2022 (weighted *n* = 77,052)

Variables of knowledge and attitude to HIV PrEP	Weighted frequency	Weighted percentage
Maternal age		
15–24	29,287	38.01
25–34	23,702	30.76
35–49	24,064	31.23
Marital status		
Never married	23,063	29.93
Married	33,941	44.05
Divorced/widowed	20,048	26.02
Maternal education		
Not educated	21,615	28.05
Primary	21,239	27.56
Secondary/higher	34,198	44.38
Wealth		
Poorest	12,162	15.78
Poorer	13,465	17.48
Middle	14,924	19.37
Richer	17,081	22.17
Richest	19,421	25.20
Heard about STIs		
No	26,050	33.81
Yes	51,002	66.19
Maternal employment		
No	30,887	40.09
Yes	46,165	59.91
Mass media exposure		
No	29,078	37.74
Yes	47,974	62.26
Knowledge modern method		
No	2,329	3.02
Yes	74,723	96.98
Number of health facility visit in 12 months		
No	34,217	44.41
Yes	42,835	55.59
At least one ANC visit		
No	8,171	1.64
Yes	68,881	98.36
Place of delivery		
Home	3,243	4.21
Health facility	73,809	95.79
Gave birth		
No	21,782	28.27
Yes	55,270	71.73
Number of under five children		
No	27,048	35.10
Yes	50,004	64.90
Current breastfeeding status		
No	60,895	79.03
Yes	16,157	20.97
Number of sex partner		
One	27,849	36.14
> One	49,203	63.86
Household head		
Male	54,530	70.77
Female	22,522	29.23
Residence		
Urban	34,754	45.10
Rural	42,298	54.90
Community ANC		
Low	39,413	51.41
High	37,639	48.59
Community institutional delivery coverage		
Low	20,681	21.14
High	56,641	78.86
Community mass media		
Low	38,837	50.50
High	38,215	49.50
Community wealth		
Low	40,277	52.79
High	36,775	47.21
Community illiteracy		
Low	37,102	47.73
High	39,950	52.27

### Knowledge, and attitude to HIV PrEP among women

The overall proportion of knowledge, and attitude to HIV PrEP to prevent getting HIV infection among women was about 13.88 (95% CI: 13.64,14.12). The highest (34.29%) and lowest (5.61%) values were observed for Kenya and Tanzania, respectively. The remaining three countries, namely Burkina Faso, Côte d’Ivoire, and Ghana, had a weighted percentage of 6.43%, 9.56%, and 12.13% respectively, indicating the prevalence of HIV PrEP in relation to HIV infection among women.

**Highlights on random effects analysis**.

The random-effects model revealed a significant clustering effect in the knowledge and attitude toward HIV PrEP for preventing HIV infection among women in different communities, with a community-level variance of 1.74. The estimated intracluster correlation coefficient (ICC) indicated that 34.46% (95% CI: 32.40, 36.57) of the variability in knowledge and attitude toward HIV PrEP was explained by variation between clusters. Individual-level factors accounted for 65.54% of the variability in women’s knowledge and attitude toward HIV PrEP. When a woman transitioned from an area with a low probability of knowledge and attitude toward HIV PrEP to an area with a high probability, there was a 51% increased probability of having better knowledge and a positive attitude toward HIV PrEP, as indicated by a MOR (Median Odds Ratio) of 3.51 (95% CI: 3.17, 3.86). The variability explained by individual-level factors, community-level factors, and both individual- and community-level factors was 34.48%, 38.51%, and 50.00%, respectively. The Likelihood Ratio (LR) test (comparing mixed-effects and logistic models, *P* = 0.00001) indicated that models considering the clustering effect provided a better fit for the data. Model IV, which incorporated both individual- and community-level factors, demonstrated the smallest deviance and largest LLR (Log-Likelihood Ratio) and was deemed the best model. The lower AIC (Akaike Information Criterion) value further supported the superiority of the final model. The associated factors were reported based on this chosen model, and therefore, the mixed-effects model was considered appropriate for analyzing the proposed topic (Table 4).

**Table 4:** Random parameters and model comparison among women from five Sub Saharan African countries demographic and health survey data, 2021–2022

Random parameters and model comparison	Null model	Model I	Model II	Model III
Community-level variance	1.74	1.14	1.07	0.87
ICC (%)	34.46	25.79	24.52	20.99
MOR (%)	3.51	2.76	2.67	2.43
PCV	Reference	34.48	38.51	50.00
Log-likelihood (LLR)	-21861.44	-20578.42	-21441.30	-112541.77
DIC (-2LLR)	43722.88	41156.84	42882.60	22509.54
AIC	43726.88	41194.84	42896.60	40902.82

### Factors associated with knowledge, and attitude to HIV PrEP among women

Women whose age group–25–34 years old (AOR = 1.52, 95% CI:1.41,1.64), and 35–49 years old (AOR = 1.56, 95% CI:1.43,1.69) have shown better knowledge and a good attitude toward HIV PrEP to prevent HIV infection by 52% and 43%, respectively, compared to women aged from 15 to 24 years old. Similarly, women who had accomplished primary (AOR = 1.79,95% CI:1.65,1.95), and secondary/higher (AOR = 2.92, 95% CI: 2.67,3.20) educational attainments were 1.79, and 2.92 times more likely to have comparatively knowledge and attitude toward HIV PrEP than formally uneducated women, respectively. Regarding household wealth indexes, women from richer (AOR = 1.14, 95% CI:1.02,1.27), and richest (AOR = 1.21, 95% CI:1.06,1.37) households revealed a higher likelihood of positive change in having knowledge and attitude toward HIV PrEP than women from the poorest households. The odds of having good knowledge and attitude to HIV PrEP was 2.82 times higher (AOR = 1.82, 95% CI:1.65,1.99) among employed women as compared to their counter parts. Having either television, radio, or reading to newspaper exposures has shown a positive association with the outcome variable (AOR = 1.49,95% CI:1.40,1.59) more times among women exposed to media. The odds of women who had knowledge about modern contraception (AOR = 2.62, 95% CI: 1.94,3.43), at least one ANC follow-up (AOR = 1.99, 95% CI:1.47,2.69), gave birth at health institutions (AOR = 1.17, 95% CI: 1.02,1.37), and ever had given birth (AOR = 1.53, 95% CI: 1.41,1.66) were more likely to have knowledge and good attitudes about HIV PrEP to prevent HIV infections compared to their counterparts, respectively. Moreover, female household heads have shown an odd of 1.24 times more likely to have good knowledge and attitude toward HIV PrEP compared to male household heads. Women from rural areas showed a 17% decrease (AOR = 83, 95% CI: 0.76,0.89) in knowledge and good attitude than urban women. Similarly, women from communities with high ANC coverage (AOR = 1.84, 95% CI: 1.61,2.11), high community mass media exposure (AOR = 1.62, 95% CI: 1.39,1.88), and women with high community wealth levels (AOR = 1.48, 95% CI: 1.30,1.68) have increased odds of knowledge and attitudes toward HIV PrEP when compared to their counterparts, respectively. Finally, women from the high illiteracy community showed a lower likelihood of having knowledge and attitudes about HIV PrEP than their counterparts (Table 5).

**Table 5:** Individual and community-level factors associated with knowledge, and attitude of HIV PrEP among women in five Sub Saharan African countries, 2021–2022

Variables of HIV PrEP	Null model I	Model I	Model II	Model III
	AOR (95% CI)	AOR (95% CI)	AOR (95% CI)	AOR (95% CI)
Maternal age				
15–24		1		1
25–34		1.53(1.41,1.65)		1.52 (1.41,1.64) *
35–49		1.58(1.45,1.72)		1.56 (1.43,1.69) *
Maternal education				
Not educated		1		1
Primary		1.88(1.72,2.05)		1.79 (1.65,1.95) *
Secondary/higher		3.13(2.86,3.43)		2.92 (2.67,3.20) *
Wealth				
Poorest		1		1
Poorer		1.08(0.97,1.19)		1.05 (0.94,1.17)
Middle		1.09(0.98,1.21)		1.06 (0.95,1.18)
Richer		1.21(1.09,1.34)		1.14 (1.02,1.27) *
Richest		1.32(1.18,1.47)		1.21 (1.06,1.37) *
Heard about STIs				
No		1		1
Yes		1.89(1.73,2.08)		1.82 (1.65,1.99) *
Employed				
No		1		1
Yes		0.95(0.89,1.01)		0.96 (0.91,1.02)
Mass media exposure				
No		1		1
Yes		1.56(1.46,1.67)		1.49 (1.40,1.59) *
Knowledge modern method				
No		1		1
Yes		2.62(1.97,3.49)		2.58 (1.94,3.43) *
ANC visit				
No		1		1
Yes		2.16(1.59,2.93)		1.99 (1.47,2.69) *
Place of delivery				
Home		1		1
Health facility		1.21(1.04,1.41)		1.17 (1.02,1.37) *
Gave birth				
No		1		1
Yes		1.54(1.42,1.67)		1.53 (1.41,1.66) *
Number of sex partner				
One		1		1
> One		1.02(0.96,1.08)		1.02 (0.96,1.08)
Household head				
Male		1		1
Female		1.26(1.19,1.34)		1.24 (1.17,1.31) *
Residence				
Urban			1	1
Rural			0.55(0.51,0.59)	0.83 (0.76,0.89) *
Community ANC				
Low			1	1
High			2.18(1.88,2.52)	1.84 (1.61,2.11) *
Community mass media				
Low			1	1
High			2.19(1.87,2.57)	1.62 (1.39,1.88) *
Community wealth				
Low			1	1
High			1.58(1.38,1.82)	1.48 (1.30,1.68) *
Community illiteracy				
Low			1	1
High			0.55(0.46,0.64)	0.71 (0.61,0.82) *

Where *= statistically significant variables at P-value < 0.05 in the final model

## Discussion

This study aimed to investigate women’s knowledge and attitudes toward PrEP for preventing HIV infection in five countries in SSA while considering individual- and community-level factors. The final model identified several factors associated with the outcome variables. At the individual level, maternal age, education, wealth index, ANC follow-up, place of delivery, sex of the household head, knowledge about modern contraception, media exposure, and awareness of STIs were found to be independently associated with women’s knowledge and attitudes toward PrEP. In terms of community-level factors, community illiteracy, community mass media exposure, community ANC coverage, community wealth, and rural/urban residence were significantly related to women’s knowledge and attitudes toward PrEP. These findings highlight the importance of considering both individual and community contexts when examining knowledge and attitudes regarding PrEP in the study population.

In this study, older women were shown to be more knowledgeable and had a positive attitude toward HIV PrEP than adolescent women. According to the search results, there is no clear evidence that older mothers have better knowledge of and attitudes towards HIV PrEP than young females. However, a study conducted in rural Western Kenya found that there is an interest and willingness to take PrEP among a substantial minority of older individuals at an elevated risk of HIV [39]. Women and girls comprise nearly half of HIV-infected individuals globally and 20% of new infections, indicating an urgent need to optimize HIV prevention options, including PrEP, in this population [40, 41]. PrEP is highly effective in preventing HIV when taken as indicated, and there are two Food and Drug Administration (FDA)-approved daily oral medications for PrEP [42]. Thus, older mothers may have better knowledge and perspectives based on their experiences. Therefore, since the disease affects the youth more, these young females should have a better understanding of the disease and different programs should take them into consideration.

Primary and secondary/higher-level educated women have revealed a higher level of knowledge and good attitude about HIV PrEP compared to formally uneducated women. Educated mothers may have better knowledge and attitudes towards HIV pre-exposure prophylaxis (PrEP) because of their access to health information, awareness programs, and possibly higher health literacy [42]. Research suggests that health professional education plays a crucial role in increasing awareness and knowledge of PrEP for HIV prevention [43, 44]. Additionally, educational attainment is often associated with better access to healthcare services and the ability to understand and effectively utilize health information [44, 45]. This can lead to improved awareness and understanding of preventive measures, such as PrEP. Furthermore, education may also be linked to a higher socioeconomic status, which can provide better access to healthcare and health-related information [45]. Overall, the combination of access to information, awareness programs, and higher health literacy due to education may contribute to better knowledge and attitudes towards HIV PrEP among educated mothers.

Rich women, and those from rich communities may take HIV PrEP more often than poor mothers due to socioeconomic inequalities. Research has shown that wealthier individuals have better access to healthcare services including HIV testing and prevention. For example, a study in East Africa found that women of higher socioeconomic status utilized more HIV testing services than their counterparts, indicating pro-rich inequalities in prenatal HIV test service uptake [46, 47]. Another study in South Africa reported that early HIV testing appeared to be higher in the lower 40% wealth group than in the higher 40% wealth group [48]. Additionally, a study in the United States, and SSA highlighted how factors such as poverty, unemployment, and inadequate access to healthcare can contribute to women’s increased vulnerability to HIV [49–51]. These findings suggest that socioeconomic factors play a significant role in the implementation of HIV prevention measures, with wealthier individuals having better access to such services. Therefore, policies and strategies should focus on inequalities and disparities in access to healthcare.

Those who have received ANC follow-ups, delivered in health facilities, and have knowledge about modern contraception have shown a positive association for HIV PrEP knowledge and attitude as compared to their counterparts. Mothers who received ANC, delivered in health facilities, and had knowledge about modern family planning were more likely to have better knowledge and a positive attitude towards HIV PrEP than those who did not. This is because of several factors: maternal and child health visits provide opportunities for healthcare providers to educate and counsel women about HIV prevention, including the use of PrEP [52]. This education can lead to increased knowledge and awareness of PrEP [53]. Healthcare providers play a critical role in delivering PrEP in maternal health service settings. Their knowledge and positive attitudes toward PrEP can influence its acceptance and uptake among pregnant women [53, 54]. Furthermore, integration of HIV services into maternal health settings has been shown to improve the uptake and retention of services related to prevention of mother-to-child transmission (PMTCT) [55, 56]. This integration can lead to increased awareness and acceptance of HIV prevention strategies including PrEP. In summary, ANC, contraceptives, and institutional delivery provide opportunities for education, counseling, and the integration of HIV services, which contributes to increased knowledge and positive attitudes towards HIV PrEP among women.

Women who had information about STIs and mass media exposure showed a positive impact on HIV PrEP knowledge and attitude as compared to those who did not have information about STIs and mass media exposure. Exposure to mass media can increase the awareness and knowledge of HIV PrEP among women. Women’s low level of awareness of HIV PrEP has been linked to the lack of PrEP advertising in places where women seek healthcare [57]. Studies have shown that mass media campaigns can be effective in increasing knowledge and behavioral changes related to HIV prevention [58]. Social media platforms, such as television, radio, and books, other social media platforms can also be used to provide PrEP information to women [59, 60]. Therefore, mass media campaigns and social media platforms can be used to increase awareness and knowledge of PrEP among women, which can lead to better attitudes towards PrEP and increased uptake of medication. Moreover, if countries use social media according to the culture and lifestyle of their people and their level of education, they can reduce the spread of the disease by instilling preventive measures, such as PrEP.

Women from rural areas showed a lower likelihood of knowledge and attitudes about HIV PrEP than urban dwellers. Rural women in Africa may not take HIV PrEP compared to urban women due to several factors. Rural women often face challenges in accessing healthcare services, including HIV testing and prevention, due to factors such as distance to healthcare facilities, lack of transportation, and limited resources [46], however, wealthier individuals, including urban women, have better access to healthcare services, including HIV testing and prevention [47]. Rural women may struggle to afford the costs associated with PrEP such as transportation, consultation fees, and medication [46]. Furthermore, rural women may experience higher levels of stigma and social exclusion due to their HIV status, which can discourage them from seeking healthcare services and accessing PrEP [46], and have lower levels of awareness about HIV prevention methods, including PrEP, due to limited access to information and resources [46]. Similarly, integrating HIV PrEP into family planning services for women in sub-Saharan Africa has faced several challenges, including difficulties in translating policy into practice, optimizing access, uptake, and effective use among populations at risk of acquiring HIV [47, 61]. Despite these challenges, studies have shown that providing universal access to PrEP in rural settings can be associated with lower HIV incidence among persons who initiate PrEP compared with matched recent controls [62]. Efforts to improve access to HIV PrEP among rural women in Africa should focus on addressing socioeconomic inequalities, improving access to healthcare services, and addressing stigma and social exclusion.

Empowered women in Africa are more likely to receive HIV PrEP because of their increased ability to make decisions about their sexual behavior and negotiate safe sex practices. Research has shown that women’s empowerment is strongly associated with their ability to ask their partners to use a condom, refuse sex, and make decisions about their sexual behavior, which are all important factors in HIV prevention [63, 64]. Additionally, women’s empowerment has been linked to better health behaviors and outcomes, including HIV testing. This is significant in the context of SSA, where fear of a partner’s reaction is a major barrier to HIV testing in many women. Therefore, empowering women and young girls has the potential to contribute to better health outcomes and to reduce the risk of HIV transmission. The “DREAMS” initiative of the United States [65]. The Agency for International Development also aims to empower adolescent girls and young women to reduce their HIV risk through various interventions, including social asset building and reproductive healthcare [63, 64].

### Strength, and limitations of the study

The utilization of nationally representative surveys from five African nations to evaluate knowledge and attitudes toward HIV PrEP to prevent HIV infection among key segments of the population (women) and related individual- and community-level factors is a fundamental strength of this study. Therefore, we believe that our findings are applicable to these countries. Another significant strength of this study was the incorporation of various possible factors of the outcome variables using an advanced mixed-effect model approach. Furthermore, the study data were gathered using conventional and verified data-gathering procedures, which could be another strength of this study. Finally, since this study is the first in its kind, it will serve as a foundation for future researchers, policymakers, and health professionals. However, the study has the following limitations. First, a cause-and-effect link cannot be demonstrated due to the cross-sectional nature of the study. Second, there might be recall bias, because it depends on self-reported statistics. Issues including accessibility to treatment, sociocultural-related factors, issues pertaining to health professionals, and support programs were not included in the analysis. Ultimately, due to limitations in data availability and constraints, we had to depend on surveys conducted in different countries within the selected countries. Therefore, more country specific studies might reveal the real scenario. This was necessary because of the data constraints we encountered and the availability of information from various sources.

### Conclusions and the way forward

This study found that women knowledge, and attitude to HIV PrEP among five SSA countries women was low. Women’s knowledge, and attitude to HIV PrEP was determined by several individual, and community level factors. Generally, less than one-seventh of women of reproductive age have shown knowledge, and attitude to HIV PrEP. Factors like maternal age, educational attainment, wealth index, exposure to the media, knowledge of modern contraceptives, ANC, health facility delivery, hearing about STIs, employment status, sex of household heads, giving birth, community level ANC coverage, community institutional delivery, community exposure to the media, illiteracy in the community, community wealth, and place of residence were found to be associated with knowledge and attitude toward HIV PrEP to prevent HIV infection among women from five SSA countries after confounders were considered.

Thus, efforts must be made to overcome the barriers that prevent women from using HIV PrEP in order to slow the spread of HIV/AIDS. Therefore, all parties active in HIV/AIDS prevention and control, regardless of nationality, ought to take into account the aforementioned variables. There will be benefits from assisting economically disadvantaged women and improving HIV PrEP education in the workplace and educational settings. Additionally, improving maternal health services, including institutional delivery, contraception, ANC, and women’s empowerment, as well as transforming the media and seizing these changes, will increase the nation’s understanding of and attitude toward HIV PrEP. Consequently, women’s health will be preserved and the prevalence of HIV/AIDS would decline.

When HIV prevention programs are implemented, considering young women, expanding access to health coverage, involving uneducated and rural women in community-wide programs, and creating awareness skills through the mass media that respects the culture and tradition of the people, and providing free drugs in large quantities, it is possible to increase women’s knowledge and improve their attitudes. We urge other countries to add information to the national population and health survey and to conduct other studies. It is advisable to consider cultural-related variables and to explore the use of spatial epidemiology approaches.

## Data Availability

All data concerning this study are accommodated and presented in this document. The detailed data set can be freely accessible from the www. dhsprogram.com website.
